# Complete Mitochondrial (mtDNA) Genome Analysis of Economically Significant Fish *Cirrhinus cirrhosus* in Bangladesh

**DOI:** 10.3390/ijms26157473

**Published:** 2025-08-02

**Authors:** Tajmirul Huda, Md. Alamgir Kabir, Md. Golam Rabbane

**Affiliations:** 1 Fisheries Genetics and Biotechnology Laboratory, Department of Fisheries, Faculty of Biological Sciences, University of Dhaka, Dhaka 1000, Bangladesh; tajmirulhuda5680@gmail.com; 2 Aquaculture Genomics Laboratory, Department of Fisheries, Faculty of Biological Sciences, University of Dhaka, Dhaka 1000, Bangladesh

**Keywords:** *Cirrhinus cirrhosus*, genome annotation, nucleotide sequence, phylogenetic analysis

## Abstract

Complete mitochondrial DNA genome annotation of an ecologically and commercially important fish species *Cirrhinus cirrhosus* was executed with next-generation sequencing (NGS) for nucleotide and phylogenetic analyses. The findings of this study showed that the *Cirrhinus cirrhosus* mitochondrial genome contained 16,593 bp, including 13 protein-coding genes, 2 ribosomal RNA genes, 22 tRNA genes, and a D-loop region. The overall base composition was 32% adenine, 25% thiamine, 16% guanine, and 27% cytosine. This mitochondrial DNA exhibits an AT biasness, with 56% AT content in its genome. Significant fluctuations were identified in the AT and GC skew values of the *ND6* gene, indicating that the selection and mutation forces acting on this gene might be different from those acting on other genes. The Ka/Ks ratios of most protein-coding genes were less than 1, indicating very strong natural selection pressure. Phylogenetic analysis of *Cirrhinus cirrhosus* with *Cirrhinus mrigala* and *Bangana tungting* suggested a closer evolutionary relationship among these species, which might have shared a more recent common ancestor. It has been also found that the genera *Labeo* and *Cirrhinus* are not monophyletic.

## 1. Introduction

*Cirrhinus cirrhosus*, known as Mrigal carp, has contributed almost 0.45% of the total riverine fish production in Bangladesh in 2021–2022 [1]. This species plays an important role in polyculture systems [2]. They inhabit the bottom layer and feed on detritus [3]. Due to facing different challenges, including seasonal variations, ecosystem degradation, pesticide and aquatic pollution, illnesses, exotic species introduction, the elimination of breeding grounds, and illegal fishing methods, their population is in danger of extinction. The IUCN has already listed them as a vulnerable species [4]. This species is still available both at natural and artificial sources. Among them, the Padma River is one of the most important. Although the Padma River contributes a large amount to the annual production of fish in Bangladesh, the biodiversity is declining day by day due to climate change, pollution, and human-induced factors [5]. However, *Cirrhinus cirrhosus* from this river has not recently been studied regarding its mitochondrial genome sequence, a commonly utilized molecular tool in population genetic structure research.

Mitochondrial DNA (mtDNA) analysis, which contains important information about genetic diversity, species identification, and evolutionary relationships, is widely used as a tool for population genetics research because of its rapid evolution and maternal inheritance [6,7]. A more thorough study of the genetic information of this species is necessary for their proper conservation and management. Whole-mtDNA sequencing is introduced in fish genetics, as previously used techniques of sequencing are mostly dependent on the availability of databases for that species, which are sometimes difficult to obtain [8]. The next-generation sequencing (NGS) method is much more efficient in sequencing the mitochondrial nucleotide [9].

Thus, this work was carried out using next-generation sequencing (NGS) analysis to find out the mitochondrial DNA genome sequence of *Cirrhinus cirrhosus* of the Padma River. These results could be considered in order to comprehend the nucleotide and phylogenetic analyses of *Cirrhinus cirrhosus* in Bangladesh.

## 2. Results

### 2.1. Mitochondrial Genome Assembly

**Figure 1:**
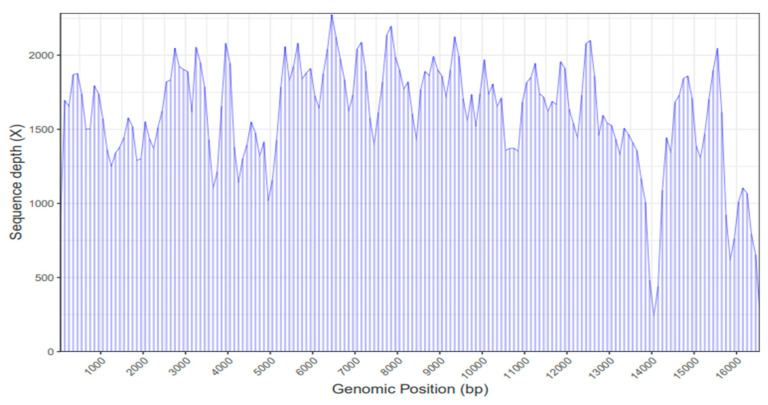
The sequencing depth of *C. cirrhosus* in terms of genome position of mitochondrial DNA.

A total of 78,977,894 reads were produced using next-generation sequencing. Low-quality reads as well as the adapter sequences were removed from the sequence. Finally, with these high-quality reads, the whole mitochondrial genome of *C. cirrhosus* was sequenced. The sequencing depth is shown in Figure 1.

**Figure 2:**
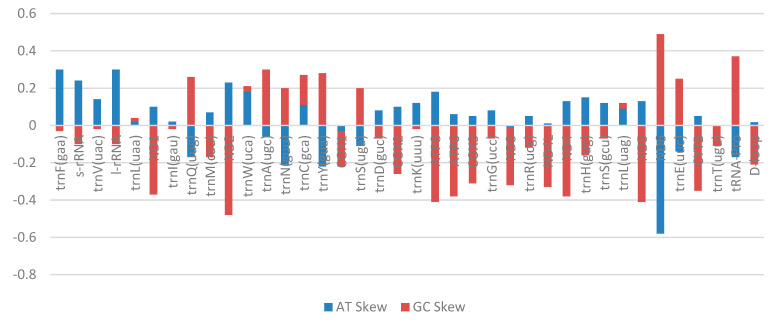
AT and GC skews of the mitogenome of *Cirrhinus cirrhosus*. Blue bars represent AT skew, and red bars represent GC skew.

**Figure 3:**
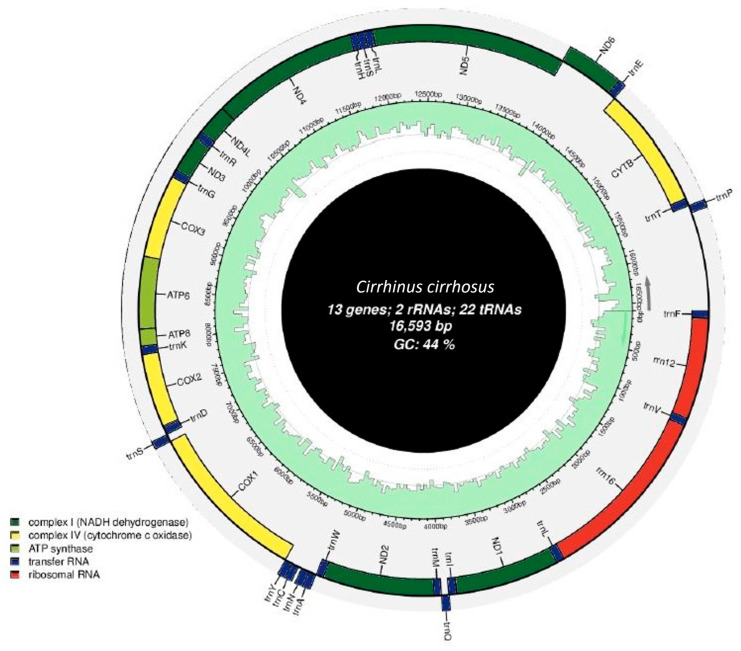
The mitochondrial genome of *Cirrhinus cirrhosus.* The organelle circle diagram is divided into three parts from the inside to the outside, including the GC content, base sequencing depth, and genetic component display. The inner circle of the gene elements is the forward-transcribed genes, and the outer circle is the reverse-transcribed genes.

The size of the mitochondrial genome of *C. cirrhosus* was 16,593 bp. It encoded 13 protein-coding genes (PGCs), 2 ribosomal RNA genes, 22 tRNA genes, and a D-loop region. It was very similar in size with the other members of the carp species. The skewness of this mitochondrial DNA was presented in Figure 2 along with a circular map was formed using the NGS data (Figure 3). There were intergenic nucleotides present in the genome of the species ranging from 1 to 33 bp, and the size of the genes ranged from 67 to 1824 bp (Table 1). The whole mitogenome of *C. cirrhosus* was submitted to GenBank (Accession no. PQ526798).

Most of the genes had higher AT content than GC content. Among them, *trnW* had the highest AT content (72%), and *trnM* had the lowest (42%). *trnV*, *trnL*, *trnM*, and *trnY* had greater GC content than AT (Table 2). trnM possessed the highest GC content (58%). The whole mitogenome of *Cirrhinus cirrhosus* had 56% AT content, which indicates the adenine and thymine richness of this species.

In the case of AT skew, the majority of the genes showed positive values, which confirmed the AT richness. Nine genes in the mitogenome (*trnQ*, *trnA*, *trnN*, *trnY*, *COX1*, *trnS*, *ND3*, *ND6*, *trnE*, and *tRNA-pro*) showed negative values in the AT skew (Table 2). Looking into the GC skew values, most of the genes were found to have negative values. Twelve genes were found with positive GC skew values, which eventually indicated GC richness. The D-loop region also showed AT richness, like the other genes.

### 2.2. Protein-Coding Genes

**Figure 4:**
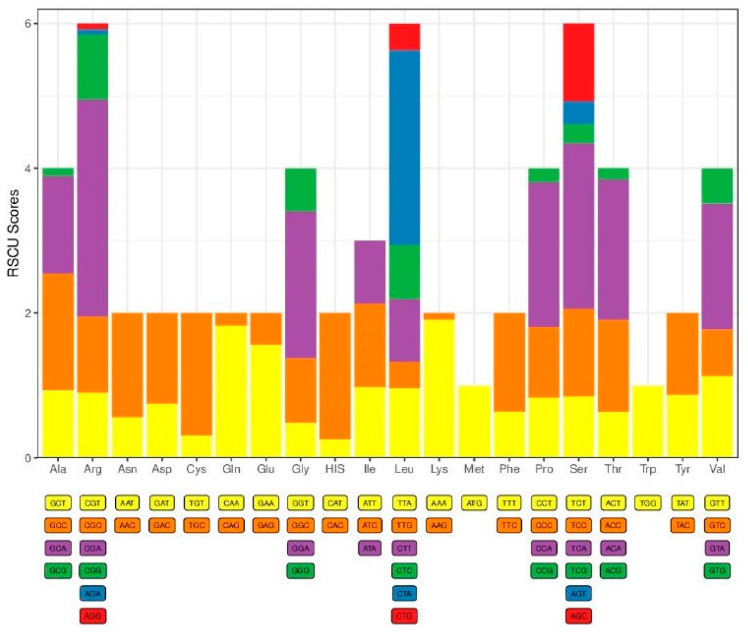
Codon bias histogram. The abscissa represents the type of amino acid translated by the codon, and the ordinate represents the codon bias score calculated for the amino acid.

There was a total of 13 protein-coding genes (PCGs) present in the genome of the experimental fish. They *were ND1*, *ND2*, *COX1*, *COX2*, *ATP8*, *ATP6*, *COX3*, *ND3*, *ND4L*, *ND4*, *ND5*, *ND6*, and *Cyt B* (Table 1). These PCGs comprised 68.8% of the total mitogenome, which was 11,410 bp in size. Most of the genes had intergenic nucleotides except for *ND2*, *COX3*, *ND3*, and *ND4L*. Among them, *ND5* (1824 bp) was the largest, and *ATP8* (165 bp) was the smallest gene. All the PCGs were forward-transcribed genes except *ND6*. This gene preferred reverse transcription. The GC content of the genome was found to be 44%, and the rest of it was AT content (Figure 3). So, it clearly shows the AT preference condition. As PCGs are the functional portion of a genome, it is common to have AT-rich content. More than one codon that coded specific amino acids was found in almost all the PCGs, but methionine and trypsin were found with only one codon for each that coded them: ATG-coded methionine and TGG-coded trypsin. The amino acids with more than one codon showed great deviations in the RSCU scores (Figure 4).

### 2.3. Transfer RNA Genes, Ribosomal RNA Genes, and Control Region

In total, 22 tRNA genes were found in the mitochondrial genome of *Cirrhinus cirrhosus* (Table 1, Figure 2). Twenty-two tRNA had a total of 1562 bp, which covered 9.4% of the whole mitogenome. Among them, *trnC*(gcc) was the shortest one and had only 67 bp (Table 1). There were two genes that had the largest sequence of 76 bp (Table 1). They were *trnL*(uaa) and *trnK*(uuu). Two rRNA genes were identified in the genome, which were *12s rRNA* and *16s rRNA* genes. *12s rRNA* (*s-rRNA*) was situated between *trnF* and *trnV* and 957 bp in size (Table 1). *16s rRNA* (*l-rRNA*), the largest *rRNA*, was found between *trnV* and *trnL* (Table 1). A non-coding control region was observed in the mitogenome of the experimental species. It is referred as the D-loop region. The D-loop had a length of 928 bp and was found between *tRNA-pro* and *trnF* (Table 1). This region had no coding functions. It is known as the control region, as it controls the replication and transcription of the genome.

### 2.4. Selective Pressure Analysis

**Figure 5:**
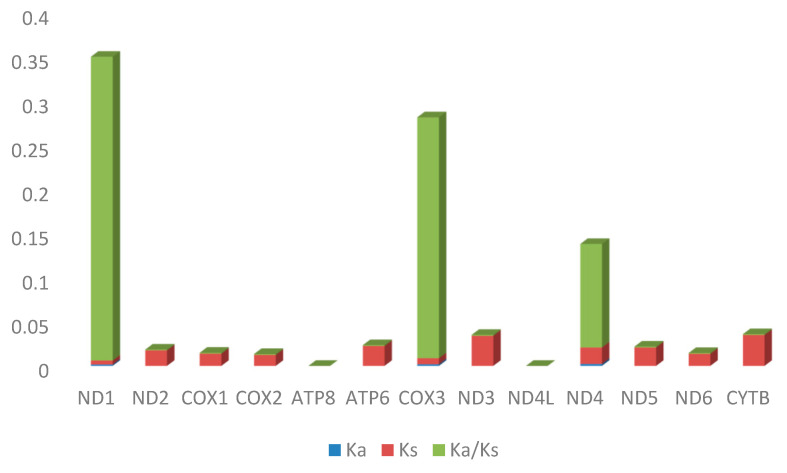
Ka, Ks, and Ka/Ks values of the 13 PGCs of *Cirrhinus cirrhosus*.

In the majority of PCGs, the Ka/Ks ratio, which is defined as the ratio of synonymous substitutions per synonymous site to non-synonymous substitutions per non-synonymous site, was much lower than 1 (Figure 5). It can be deduced from this that these genes were undergoing high levels of purifying selection, also known as negative selection. This means that non-synonymous mutations were being eliminated because of the detrimental consequences they had on the function of the protein. With both zero Ka and Ks values for the *ATP8* and *ND4L genes*, the Ka/Ks ratio was zero (Figure 5). This implies that among the 13 PCGs, these genes were highly conserved and under the most purifying selection.

The *p*-values that were related to the Ka/Ks ratios were greater for the genes *ND1*, *COX3*, *ND4*, and *ND6* (Table 3). This indicated that the ratios did not differ significantly from what would be predicted under the assumption of neutral evolution. Based on this information, it appeared that these genes may be subject to a lower level of selective pressure in comparison to the other PCGs. The *p*-values were quite small (<0.05) for the majority of the PCGs with very low Ka/Ks ratios, therefore verifying the statistically significant purifying selection observed. These results imply that most of the mitochondrial protein-coding genes in *Cirrhinus cirrhosus* were strongly under evolutionary constraints to preserve their functional integrity.

### 2.5. Nucleotide Diversity

**Figure 6:**
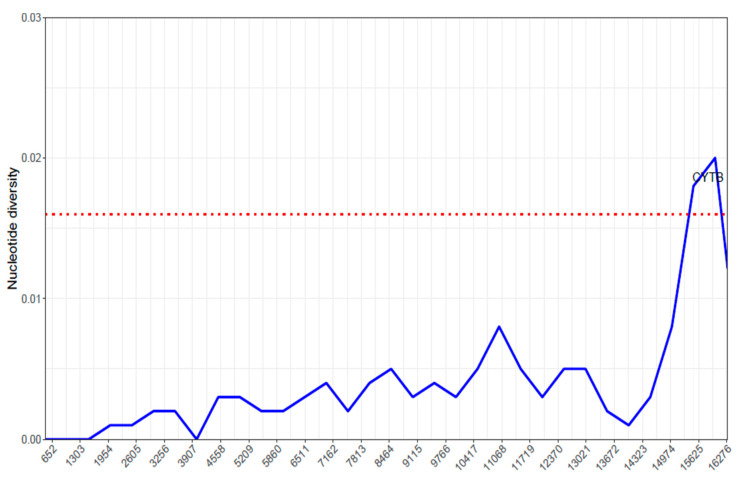
Nucleotide diversity across the mitochondrial genome of *Cirrhinus cirrhosus*.

Figure 6 indicates the nucleotide diversity across different regions of the mitochondrial genome of *Cirrhinus cirrhosus*. The whole mitochondrial genome was not similarly distributed in the sense of diversity. Various regions of the genome showed various nucleotide percentages, which indicated some conserved regions and some regions of genetic variability.

The first locations, which ranged from 650 to almost 13,000, showed generally low nucleotide diversity values, suggesting that these areas were quite conserved across the examined sequences. The nucleotide diversity showed moderate peaks at numerous places, including about 5200, 8464, and 13,021 bp, which indicated regions with increased genetic variety. These positions were located in the middle of the genome (Figure 6). Around position 15,625, a large surge in the diversity of nucleotides was discovered (Figure 6). According to this, there was a section of the genome that exhibited a significant degree of genetic diversity, which belongs to the *CYTB* gene, indicating that it might have been subject to different selective pressures or might have played a role in adaptive divergence within the species. This graph (Figure 6) revealed the various degrees of nucleotide diversity across the mitochondrial genome of *Cirrhinus cirrhosus*. Low-diversity regions indicated evolutionary conservation, while higher-diversity regions, especially the *CYTB* gene, highlighted areas of genetic heterogeneity and potential adaptive relevance.

### 2.6. Phylogenetic Analysis

**Figure 7:**
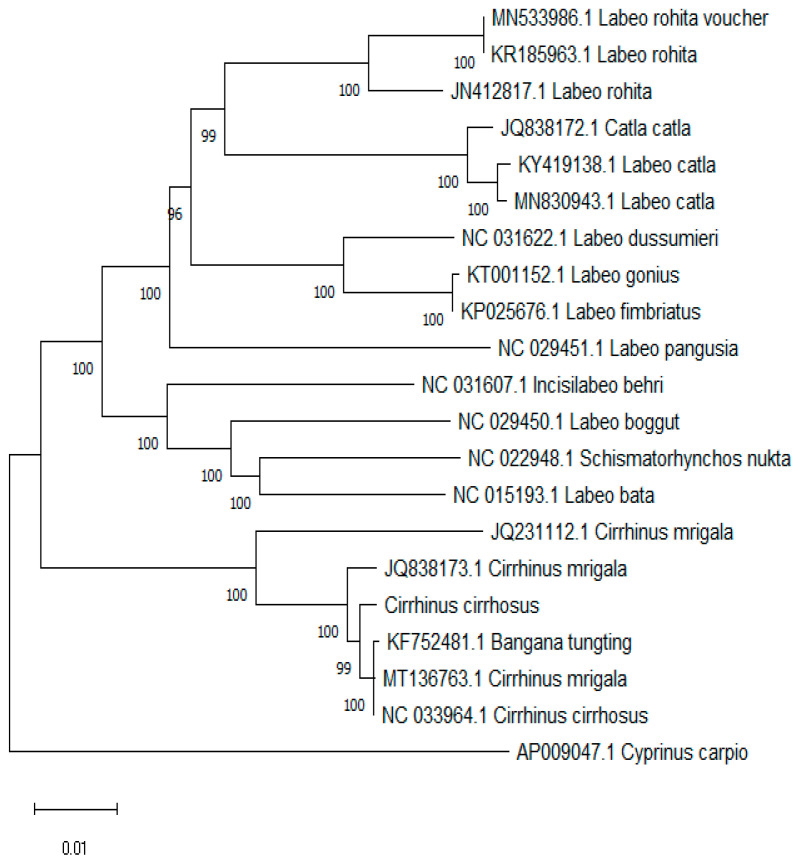
Phylogenetic relationship of the Cyprinidae family.

Starting with a common ancestor, the phylogenetic tree traced different species from which branches emerged. With bootstrap values of 100, the *Labeo rohita* species (MN533506.1; KR185963.1) developed a clear clade, showing rather high confidence in their tight evolutionary relationship (Figure 7). Strong support for their grouping was indicated by the clade comprising *Catla catla* (JQ838172.1; KY419138.1), with a 100-bootstrap value. Strong support with bootstrap values of 100 was shown by the clade including *Labeo dussumieri* (NC031622.1), *Labeo gonius* (KT001152.1), and *Labeo fimbriatus* (KP025676.1). *Labeo pangusia* (NC029451) generated a clear branch. Separated on a different branch, *Incisilabeo behri* (NC031607.1) indicated its unique evolutionary background inside the group (Figure 7).

With *Bangana tungting* (KF752481.1) and *Cirrhinus mrigala* (MT136763.1), *Cirrhinus cirrhosus* (NC0 33964.1) created a clade displaying bootstrap values of 91 and 99, thereby demonstrating great confidence in their tight association (Figure 7). Comparatively to the other species in the tree, the close grouping of *Cirrhinus cirrhosus* with *Cirrhinus mrigala* and *Bangana tungting* revealed a closer evolutionary link among these species, which might have shared a more recent common ancestor. The tree included *Cyprinus carpio* (AP009047.1) as an outgroup, which helped root the tree and provided a reference point (Figure 7). The close clustering of *Cirrhinus cirrhosus* with *Cirrhinus mrigala* and *Bangana tungting* suggested a closer evolutionary relationship among these species, which might have shared a more recent common ancestor compared to the other species in the tree. There were multiple sequences present for the one species in the NCBI, which formed different clades despite being the same species. The analysis showed that the genera *Labeo* and *Cirrhinus* are not monophyletic, as they formed clades with different species.

## 3. Discussion

The complete mitogenome of *Cirrhinus cirrhosus* (16,593 bp) was quite similar to other carp species like *Cyprinus carpio* (16,606 bp) and *Carassius auratus* (16,580 bp) [10,11]. The mitogenome also contains 13 protein-coding genes, 2 ribosomal RNA genes, 22 tRNA genes, and a D-loop region that is consistent with the structure of other teleost fish mitogenomes [12,13]. In addition, the presence of intergenic nucleotides ranging from 1 to 33 base pairs and gene sizes ranging from 67 to 1824 base pairs are consistent with the compact character of vertebrate mitochondrial genomes [13].

The *C. cirrhosus* mitogenome is AT rich, which covers 56% of the whole mitogenome, with most genes displaying positive AT skew values. It is most likely a result of the high metabolic rate and body temperature of this species, as it is found in the subcontinent [14]. Most of the *tRNA genes* have more AT content than GC. Reportedly due, in part, to adenine’s function in preserving the tRNA secondary structure, the AT-rich region is especially strong in *tRNA*s [11]. The asymmetric character of mitochondrial DNA replication and transcription is supposed to affect this pattern by producing an excess of guanine in the coding strand [15]. It is hypothesized that the varying mutation rates experienced by the leading and lagging strands during replication produce the positive GC skew [15]. Consistent with its function in promoter activity, the D-loop region is responsible for the control of mitochondrial DNA replication and transcription and also exhibits an AT richness [13]. *C. cirrhosus* and other carp species have similar mitogenome structures and nucleotide contents, which points to a shared evolutionary past inside the family Cyprinidae.

The content of the genes and organization are remarkably conserved. Except for *ND2*, *COX3*, *ND3*, and *ND4L*, most of the protein-coding genes have intergenic nucleotides between them. These intergenic sections’ varying lengths, from 1 to 33 base pairs, help to define the mitogenome’s compact character. Reflecting the variety in gene sizes within the mitochondrial genome, *ND5*, the biggest protein-coding gene, is 1824 bp in length; the smallest, *ATP8*, is just 165 bp. Except for *ND6*, which is reverse transcribed, the main forward transcription of the protein-coding genes is a normal feature of vertebrate mitochondrial genomes [16]. The strand-specific mutation patterns seen in mitochondrial DNA are hypothesized to affect this asymmetric transcription [15]. The *C. cirrhosus* mitogenome has 44% total GC content; the remaining 56% is AT content. As reported in other teleost fishes [17] and reptiles [18], the preference for AT-rich codons in the protein-coding genes is in line with this genome-wide AT bias. Like other teleost fish, a total 22 *tRNA genes* were identified in the mitochondrial genome of *C. cirrhosus* [13]. Through their delivery of amino acids to the ribosome during translation, these tRNA genes are essential for mitochondrial protein synthesis [19]. Consistent with the results in other fish mitochondrial genomes, the variance in *tRNA* gene lengths—*trnC*(gcc) being the smallest at 67 base pairs and *trnL*(uaa) and *trnK*(uuu) being the longest at 76 base pairs—is clear cut. There were two highly conserved *rRNA* genes found in the *C. cirrhosus* mitogenome: *12S rRNA* (*s-rRNA*) and *16S rRNA* (*l-rRNA*). Both small and large ribosomal subunits depend on these *rRNA genes*, which also are required for mitochondrial protein synthesis [20]. While the *16S rRNA* gene between *trnV* and *trnL* helps to generate the large ribosomal subunit, the *12S rRNA* gene between *trnF* and *trnV* is in charge of the structural integrity of the small ribosomal subunit [20]. The D-loop, a region also referred to as the control region of the mitochondrial genome of *C. cirrhosus*, is similar to other vertebrates. It is a non-coding, regulating sequence. Important regulating factors in these 928 base-pair regions between *tRNA-Pro* and *trnF* include sites for transcription initiation and the origin of replication [21]. Particularly in teleost fishes, the inclusion of 22 *tRNA genes*, 2 *rRNA genes*, and a D-loop region in the mitochondrial genome of *C. cirrhosus* conforms with the usual organization of vertebrate mitochondrial genomes [13].

The Ka/Ks ratio, also known as the dN/dS ratio, is a frequently used statistic to quantify the mechanism and degree of selection operating on coding sequences [22]. In the majority of PCGs, the Ka/Ks ratio was significantly lower than 1, indicating that these genes were receiving strong levels of purifying selection [23]. Purifying selection, also known as negative selection, serves to eliminate non-synonymous mutations that are deleterious to the function of the encoded protein [24]. These data show that most mitochondrial protein-coding genes in *C. cirrhosus* are under substantial evolutionary constraints to maintain their functional integrity. The *ATP8* and *ND4L* genes both have zero Ka and Ks values, resulting in a Ka/Ks ratio of zero. This means that these genes are highly conserved and under the most intensive purifying selection among the 13 PCGs [25]. The remarkable conservation of these genes may be owing to their crucial involvement in the functioning of the mitochondrial electron transport chain and ATP production [21]. The *p*-values linked with the Ka/Ks ratios for the genes *ND1*, *COX3*, *ND4*, and *ND6* were higher. This implies that among the other PCGs, these genes might be susceptible to less selective pressure [25]. The great purifying selection operating on most protein-coding genes and the vital roles these areas play in mitochondrial function most certainly contribute to this conservation [26]. The most obvious aspect of the nucleotide diversity plot is the significant increase in diversity near position 15,625, which relates to the *Cyt B* gene. This result implies that the *Cyt B* gene might have been subject to various selective pressures or might have contributed to adaptive divergence within *C. cirrhosus* [27].

The tree illustrated that *Labeo rohita* formed a well-supported clade, which was strongly supported by bootstrap values of 100. This high confidence indicated a close evolutionary relationship among the different *Labeo rohita* samples (MN533506.1; KR185963.1). Similarly, the *Catla catla* clade also exhibited strong support, confirming the tight evolutionary relationship among the samples (JQ838172.1; KY419138.1). This indicated that *Catla catla* maintained genetic consistency across different populations, reflecting stable evolutionary traits within this species. The clade comprising *Labeo dussumieri*, *Labeo gonius*, and *Labeo fimbriatus* also showed robust support. This strong bootstrap value underscored the close genetic relationships and possible shared evolutionary paths among these species. The distinct branch formation by *Labeo pangusia* indicated its clear genetic demarcation from other species, suggesting unique evolutionary adaptations or historical separation events. Interestingly, *Incisilabeo behri* formed a separate branch, indicating its unique evolutionary lineage within the group. This separation pointed to distinct genetic characteristics and potential adaptive differences. In another well-supported clade, *Cirrhinus mrigala*, *Cirrhinus cirrhosus*, and *Bangana tungting* formed a cluster with bootstrap values of 91 and 99, respectively. This clade revealed a close evolutionary relationship among these species, suggesting they might have shared a recent common ancestor. The clustering indicated that these species underwent recent evolutionary divergence, possibly driven by similar environmental pressures or geographical proximities. The positioning of *Cyprinus carpio* as an outgroup allowed for clearer insights into the divergence and evolutionary pathways of the Cyprinidae family members.

The findings of the study could contribute to the management and protection of mitogenome resources of *C. cirrhosus*, as well as development of molecular techniques for species identification for the preservation and sustainable growth of aquaculture in Bangladesh. Furthermore, mitogenome characterization of wild *C. cirrhosus* collected from the Padma River will be a valuable and precious resource in molecular evolutionary biology for improving commercially important native species in Bangladesh. To create stronger and more reliable relationships with mitogenomic and other genetic markers, it is recommended to expand the sample size and include more molecular marker information, such as nuclear DNA, microsatellites, minisatellites, AFLP, RFLP, RAPD, and morphology. Also, effective initiatives should be taken to compare the mitogenomic diversities of different important aquatic habitats, such as different rivers or lakes, to understand their genetic evolution. In order to conserve and manage this commercially significant fish species effectively, these findings should be applied for species identification, diversity assessment, and genetic connection analysis.

## 4. Materials and Methods

### 4.1. Ethics Statement

All authors declare that all experiments have been ethically examined and approved by the Ethical Review and Clearance Committee of the Faculty of Biological Sciences, University of Dhaka, with the ethical clearance certificate numbered as Ref. No. 285/Biol.Scs and dated 26 September 2024.

### 4.2. Sample Collection, DNA Extraction, and Quality Assessment

The *Cirrhinus cirrhosus* fish were collected from the Padma River of Paba Upazila in Rajshahi district (88°30′42.157″ E–24°21′51.348″ N), Bangladesh, from May to June 2024. The live fish species was transported to the Fisheries Genetics and Biotechnology Laboratory, Department of Fisheries, University of Dhaka. After species confirmation morphometrically, the tissue samples were collected primarily from the muscle with sharp sterile scissors. The pooled 25 mg of muscle tissue was used to extract DNA using an automated DNA extractor (model: Maxwell 16; origin: Promega, Madison, WI, USA) according to manufacturer’s guidelines. Three hundred microliters of nuclease-free water were used to homogenize the tissue samples. The elution tube was then filled with 300 μL of elution buffer. For 35 min, DNA kits were washed in the Maxwell^®^ 16 MDx Research Instrument (Promega, USA) Automatic Nucleic Acid Purification System. Then, the samples were preserved at −20 °C in a freezer for further analysis.

### 4.3. Next-Generation Sequencing

For sequencing, high-quality genomic DNA was cut into smaller fragments, which was followed by end repair, adapter ligation, PCR amplification, and quality assessment. To sequence on the Illumina HiSeq 2500 platform (Illumina, San Diego, CA, USA), the pooled libraries were denatured and diluted to the suitable loading concentration. The prepared library pool was put onto the flow cell. Cluster formation was conducted using the Illumina cBot system, which generated clusters of identical DNA fragments on the surface of the flow cell. Sequencing was performed on the Illumina HiSeq 2500 platform, producing 150 bp paired-end reads. The sequencing run was completed according to the manufacturer’s specifications, assuring great coverage and accuracy. Fastp v0.36 was originally used to evaluate the raw sequencing readings taken for a quality test. The cleaned reads were aligned to a reference mitochondrial genome using Bowtie2 v2.1.0.

### 4.4. Mitogenome Assembly and Annotation

The aligned reads were assembled into contigs using SPAdes v3.15, which is particularly suitable for assembling short-read sequences. For comparison, assembly was also performed using megahit v1.2.9, which is optimized for large and complex metagenomic data sets. The quality of the assembled contigs was evaluated using QUAST (Quality Assessment Tool for Genome Assemblies). The initial contigs generated by SPAdes and megahit were further processed to close gaps and extend contigs using GapFiller v1.11. The final assembly of the mitochondrial genome was refined using GetOrganelle v1.7.5.3, which specifically targets organellar genomes. The assembled mitochondrial genome was annotated by aligning it to known mitochondrial sequences using NCBI BLAST+ v2.28. The BLAST results were used to identify conserved genes and other functional elements in the mitochondrial genome. Simple Sequence Repeats (SSRs) were identified in the mitochondrial genome using misa v2.1. The rates of synonymous (Ks) and non-synonymous (Ka) substitutions were calculated using KaKs Calculator v3.0. AT and GC skew values were calculated using the following formulas: AT skew = (A − T)/(A + T) and GC skew = (G − C)/(G + C).

### 4.5. Phylogenetic Analysis

The mitochondrial genome sequences of *Cirrhinus cirrhosus* and closely related species were aligned using ClustalW 2.1 to prepare for phylogenetic tree construction. MEGA 11 was employed to construct maximum-likelihood phylogenetic trees using the best-fit model. Bootstrap values were taken in several thousands to have more accurate results. A total of 21 sequences were used in the phylogenetic analysis of this study. Among them, one is the sequence achieved from the study, and the others were collected from the NCBI database to understand the phylogenetic relationship of the studied species with other species from the same family.

## 5. Conclusions

The findings of this study showed that the *C. cirrhosus* mitochondrial DNA genome is similar with other vertebrate mitochondrial genomes. This genome annotation and phylogenetic information will provide a baseline for further proper conservation and management of this important fish species.

## Figures and Tables

**Table 1:** Gene annotations of the complete mitochondrial genome of Cirrhinus cirrhosus.

Gene	Gene Type	Location	Intergenic Nucleotides	Size
*trnF*(*gaa*)	tRNA	1–69	0	69
*s-rRNA*	rRNA	70–1026	0	957
*trnV*(*uac*)	tRNA	1027–1098	0	72
*l-rRNA*	rRNA	1099–2786	0	1688
*trnL*(*uaa*)	tRNA	2787–2862	0	76
*ND1*	CDS	2864–3838	1	975
*trnI*(*gau*)	tRNA	3843–3914	4	72
*trnQ*(*uug*)	tRNA	3913–3983	−2	71
*trnM*(*cau*)	tRNA	3985–4053	1	69
*ND2*	CDS	4054–5098	0	1045
*trnW*(*uca*)	tRNA	5099–5169	0	71
*trnA*(*ugc*)	tRNA	5172–5240	2	69
*trnN*(*guu*)	tRNA	5242–5314	1	73
*trnC*(*gca*)	tRNA	5348–5414	33	67
*trnY*(*gua*)	tRNA	5415–5484	0	70
*COX1*	CDS	5486–7036	1	1551
*trnS*(*uga*)	tRNA	7037–7107	0	71
*trnD*(*guc*)	tRNA	7111–7182	3	72
*COX2*	CDS	7196–7886	13	691
*trnK*(*uuu*)	tRNA	7887–7962	0	76
*ATP8*	CDS	7964–8128	1	165
*ATP6*	CDS	8122–8804	−7	683
*COX3*	CDS	8805–9590	0	786
*trnG*(*ucc*)	tRNA	9591–9662	0	72
*ND3*	CDS	9663–10,011	0	349
*trnR*(*ucg*)	tRNA	10,012–10,081	0	70
*ND4L*	CDS	10,082–10,378	0	297
*ND4*	CDS	10,372–11,752	−7	1381
*trnH*(*gug*)	tRNA	11,753–11,821	0	69
*trnS*(*gcu*)	tRNA	11,822–11,890	0	69
*trnL*(*uag*)	tRNA	11,892–11,964	1	73
*ND5*	CDS	11,968–13,791	3	1824
*ND6*	CDS	13,788–14,309	−4	522
*trnE*(*uuc*)	tRNA	14,310–14,378	0	69
*CYTB*	CDS	14,384–15,524	5	1141
*trnT*(*ugu*)	tRNA	15,525–15,596	0	72
*tRNA-Pro*	tRNA	15,596–15,665	−1	70
D-loop	misc_feature	15,666–16,593	0	928

**Table 2:** Nucleotide compositions of mitochondrial genes of *Cirrhinus cirrhosus*.

Gene	Total Bases	Number of Individual Bases				Percentage of Bases (%)				Percentage Content		AT Skew	GC Skew
		A	T	G	C	A	T	G	C	AT%	GC%		
*trnF*(*gaa*)	69	26	14	14	15	37	22	20	21	59	41	0.3	−0.03
*s-rRNA*	957	305	184	210	258	31	22	21	26	53	47	0.24	−0.10
*trnV*(*uac*)	72	20	15	18	19	27	22	25	26	49	51	0.14	−0.02
*l-rRNA*	1688	624	334	328	402	36	22	19	23	58	42	0.30	−0.10
*trnL*(*uaa*)	76	19	18	20	19	25	24	26	25	49	51	0.02	0.02
*ND1*	975	292	235	141	307	29	26	14	31	55	45	0.10	−0.37
*trnI*(*gau*)	72	19	18	17	18	26	26	23	25	52	48	0.02	−0.02
*trnQ*(*uug*)	71	17	24	19	11	23	36	26	15	59	41	−0.17	0.26
*trnM*(*cau*)	69	15	13	17	24	21	21	24	34	42	58	0.07	−0.17
*ND2*	1045	353	218	121	353	33	23	11	33	56	44	0.23	−0.48
*trnW*(*uca*)	71	26	18	14	13	36	27	19	18	63	37	0.18	0.03
*trnA*(*ugc*)	69	23	26	13	7	33	39	18	10	72	28	−0.06	0.3
*trnN*(*guu*)	73	15	23	21	14	20	33	28	19	53	47	−0.21	0.2
*trnC*(*gca*)	67	20	16	18	13	29	26	26	19	55	45	0.11	0.16
*trnY*(*gua*)	70	12	19	25	14	17	28	35	20	45	55	−0.22	0.28
*COX1*	1551	416	446	277	412	26	31	17	26	57	43	−0.03	−0.19
*trnS*(*uga*)	71	16	20	21	14	22	30	29	19	52	48	−0.11	0.2
*trnD*(*guc*)	72	25	21	12	14	34	31	16	19	65	35	0.08	−0.07
*COX2*	691	215	175	111	190	31	26	16	27	57	43	0.10	−0.26
*trnK*(*uuu*)	76	23	18	17	18	30	25	22	23	55	45	0.12	−0.02
*ATP8*	165	59	41	19	46	35	27	11	27	62	38	0.18	−0.41
*ATP6*	683	208	184	90	201	30	28	13	29	58	42	0.06	−0.38
*COX3*	786	224	199	125	238	28	27	15	30	55	45	0.05	−0.31
*trnG*(*ucc*)	72	25	21	12	14	34	31	16	19	65	35	0.08	−0.07
*ND3*	349	96	98	53	102	27	29	15	29	56	44	−0.01	−0.31
*trnR*(*ucg*)	70	20	18	14	18	28	27	20	25	55	45	0.05	−0.12
*ND4L*	297	78	75	48	96	26	26	16	32	52	48	0.01	−0.33
*ND4*	1381	449	345	181	406	32	26	13	29	58	42	0.13	−0.38
*trnH*(*gug*)	69	26	19	10	14	37	29	14	20	66	34	0.15	−0.16
*trnS*(*gcu*)	69	23	18	13	15	33	28	18	21	61	39	0.12	−0.07
*trnL*(*uag*)	73	23	19	16	15	31	28	21	20	59	41	0.09	0.03
*ND5*	1824	612	463	220	529	33	26	12	29	59	41	0.13	−0.41
*ND6*	522	59	227	176	60	11	45	33	11	56	44	−0.58	0.49
*trnE*(*uuc*)	69	18	24	17	10	26	36	24	14	62	38	−0.14	0.25
*CYTB*	1141	339	305	161	336	29	28	14	29	57	43	0.05	−0.35
*trnT*(*ugu*)	72	18	18	16	20	25	26	22	27	51	49	0	−0.11
*tRNA-Pro*	70	17	24	20	9	24	36	28	12	60	40	−0.17	0.37
D-loop	928	320	309	117	182	34	35	12	19	69	31	0.017	−0.21

**Table 3:** Ka/Ks statistics table.

Sequence	Ka	Ks	Ka/Ks	*p*-Value (Fisher)	Substitutions	Syn-Subs	Non-syn-Subs
*ND1*	0.00155	0.004498	0.344636	0.580742	2	0.882608	1.11739
*ND2*	1.75 × 10^−5^	0.017535	0.001	0.001957	3	2.98596	0.014036
*COX1*	1.39 × 10^−5^	0.013891	0.001	0.000623	4	3.98442	0.01558
*COX2*	1.25 × 10^−5^	0.012476	0.001	0.023376	2	1.9941	0.005899
*ATP8*	0	0	0	0	0	0	0
*ATP6*	2.26 × 10^−5^	0.022563	0.001	0.002104	4	3.98957	0.010428
*COX3*	0.001867	0.006831	0.273377	0.54937	2	0.922892	1.07711
*ND3*	3.42 × 10^−5^	0.034179	0.001	0.00688	3	2.99182	0.008185
*ND4L*	0	0	0	0	0	0	0
*ND4*	0.002198	0.018689	0.117584	0.005217	7	4.81737	2.18263
*ND5*	2.09 × 10^−5^	0.020923	0.001	3.87 × 10^−6^	7	6.97138	0.028618
*ND6*	1.37 × 10^−5^	0.013684	0.001	0.053704	1	0.994184	0.005816
*CYTB*	3.50 × 10^−5^	0.035013	0.001	1.40 × 10^−6^	8	7.9705	0.029501

## Data Availability

The whole mitogenome of *C. cirrhosus* are available in GenBank (Accession no. PQ526798). https://www.ncbi.nlm.nih.gov/nuccore/PQ526798.

## References

[1] DoF (2022). Yearbook of Fisheries Statistics of Bangladesh, 2021–2022.

[2] Biswas G., Jena J.K., Singh S.K., Patmajhi P., Muduli H.K. (2006). Effect of feeding frequency on growth, survival and feed utilization in mrigal, *Cirrhinus mrigala*, and rohu, *Labeo rohita*, during nursery rearing. Aquaculture.

[3] Chauhan T., Lal K.K., Mohindra V., Singh R.K., Punia P., Gopalakrishnan A., Sharma P.C., Lakra W.S. (2007). Evaluating genetic differentiation in wild populations of the Indian major carp, *Cirrhinus mrigala* (Hamilton-Buchanan, 1882): Evidence from allozyme and microsatellite markers. Aquaculture.

[4] (2015). UCN Bangladesh Red List of Bangladesh Volume 1: Summary.

[5] Aktera R., Sharifb S.H., Khanc M.R. (2022). An overview of fish biodiversity and socioeconomic situation of the Padma river’s fisher community in Bangladesh. Big Data Agric. (BDA).

[6] Jahan H., Chakraborty M., Alam M.S., Begum R.A. (2024). Characterization of complete mitochondrial genome of *Labeo rohita* from Bangladesh. Bioresearch Commun. (BRC).

[7] Singh M., Saini V.P., Mohindra V., Ojha M.L., Lal K.K., Singh R.K. (2023). Complete mitochondrial genome of golden variant of freshwater fish *Labeo rajasthanicus* (Cypriniformes: Cyprinidae): Endemic to India. Mitochondrial DNA Part B.

[8] Schroeter J.C., Maloy A.P., Rees C.B., Bartron M.L. (2020). Fish mitochondrial genome sequencing: Expanding genetic resources to support species detection and biodiversity monitoring using environmental DNA. Conserv. Genet. Resour..

[9] Zhang R., Zhu T., Luo Q. (2023). The Complete Mitochondrial Genome of the Freshwater Fish *Onychostoma ovale* (Cypriniformes, Cyprinidae): Genome Characterization and Phylogenetic Analysis. Genes.

[10] Ye X., Lv Y., Wei L., Huang J., Wen Y., Zhang G., Zhang S., Yang Z., Liu K. (2018). The complete mitochondrial genome of Jinbian carp *Cyprinus carpio* (Cypriniformes: Cyprinidae). Mitochondrial DNA Part B.

[11] Zhou C., Li B., Ma L., Zhao Y., Kong X. (2014). The complete mitogenome of natural triploid *Carassius auratus* in Qihe River. Mitochondrial DNA Part A.

[12] Luo L., Xu Y., Wang S., Zhang R., Guo K., Xu W., Zhao Z. (2023). Complete Mitochondrial Genome Sequence and Phylogenetic Analysis of *Procambarus clarkii* and *Cambaroides dauricus* from China. Int. J. Mol. Sci..

[13] Satoh T.P., Miya M., Mabuchi K., Nishida M. (2016). Structure and variation of the mitochondrial genome of fishes. BMC Genom..

[14] Joseph J., Sreeedharan S., George S., Antony M.M. (2022). The complete mitochondrial genome of an endemic cichlid *Etroplus canarensis* from Western Ghats, India (Perciformes: Cichlidae) and molecular phylogenetic analysis. Mol. Biol. Rep..

[15] Reyes A., Gissi C., Pesole G., Saccone C. (1998). Asymmetrical directional mutation pressure in the mitochondrial genome of mammals. Mol. Biol. Evol..

[16] Desjardins P., Morais R. (1990). Sequence and gene organization of the chicken mitochondrial genome: A novel gene order in higher vertebrates. J. Mol. Biol..

[17] Chang Y.-S., Huang F.-L., Lo T.-B. (1994). The complete nucleotide sequence and gene organization of carp (*Cyprinus carpio*) mitochondrial genome. J. Mol. Evol..

[18] Montaña-Lozano P., Balaguera-Reina S.A., Prada-Quiroga C.F. (2023). Comparative analysis of codon usage of mitochondrial genomes provides evolutionary insights into reptiles. Gene.

[19] Temperley R.J., Wydro M., Lightowlers R.N., Chrzanowska-Lightowlers Z.M. (2010). Human mitochondrial mRNAs—Like members of all families, similar but different. Biochim. Et Biophys. Acta (BBA)-Bioenerg..

[20] Sharma M.R., Koc E.C., Datta P.P., Booth T.M., Spremulli L.L., Agrawal R.K. (2003). Structure of the mammalian mitochondrial ribosome reveals an expanded functional role for its component proteins. Cell.

[21] Fernández-Silva P., Enriquez J.A., Montoya J. (2003). Replication and transcription of mammalian mitochondrial DNA. Exp. Physiol..

[22] Kryazhimskiy S., Plotkin J.B. (2008). The population genetics of dN/dS. PLoS Genet..

[23] Kimura M. (1977). Preponderance of synonymous changes as evidence for the neutral theory of molecular evolution. Nature.

[24] Nei M., Gojobori T. (1986). Simple methods for estimating the numbers of synonymous and nonsynonymous nucleotide substitutions. Mol. Biol. Evol..

[25] Nei M., Kumar S. (2000). Molecular Evolution and Phylogenetics.

[26] Nabholz B., Glémin S., Galtier N. (2013). The erratic mitochondrial clock: Variations of mutation rate, not population size, affect mtDNA diversity across birds and mammals. BMC Evol. Biol..

[27] Nabholz B., Glémin S., Galtier N. (2008). Strong variations of mitochondrial mutation rate across mammals—The longevity hypothesis. Mol. Biol. Evol..

